# Response to Alternating Electric Fields of Tubulin Dimers and Microtubule Ensembles in Electrolytic Solutions

**DOI:** 10.1038/s41598-017-09323-w

**Published:** 2017-08-29

**Authors:** Iara B. Santelices, Douglas E. Friesen, Clayton Bell, Cameron M. Hough, Jack Xiao, Aarat Kalra, Piyush Kar, Holly Freedman, Vahid Rezania, John D. Lewis, Karthik Shankar, Jack A. Tuszynski

**Affiliations:** 1 Department of Electrical & Computer Engineering, University of Alberta, Edmonton Alberta, T6G 1H9 Canada; 2 Department of Oncology, University of Alberta, Edmonton Alberta, T6G 1Z2 Canada; 3 NRC National Institute for Nanotechnology, Edmonton Alberta, T6G 2M9 Canada; 4 Department of Physics, University of Alberta, Edmonton Alberta, T6G 2E1 Canada; 5 Department of Medical Physics, Cross Cancer Institute, Edmonton Alberta, T6G 1Z2 Canada; 6 Department of Medical Microbiology and Immunology, University of Alberta, Edmonton Alberta, T6G 2E1 Canada; 7 Department of Physical Sciences, MacEwan University, Edmonton Alberta, T5J 4S2 Canada

## Abstract

Microtubules (MTs), which are cylindrical protein filaments that play crucial roles in eukaryotic cell functions, have been implicated in electrical signalling as biological nanowires. We report on the small-signal AC (“alternating current”) conductance of electrolytic solutions containing MTs and tubulin dimers, using a microelectrode system. We find that MTs (212 nM tubulin) in a 20-fold diluted BRB80 electrolyte increase solution conductance by 23% at 100 kHz, and this effect is directly proportional to the concentration of MTs in solution. The frequency response of MT-containing electrolytes exhibits a concentration-independent peak in the conductance spectrum at 111 kHz (503 kHz FWHM that decreases linearly with MT concentration), which appears to be an intrinsic property of MT ensembles in aqueous environments. Conversely, tubulin dimers (42 nM) decrease solution conductance by 5% at 100 kHz under similar conditions. We attribute these effects primarily to changes in the mobility of ionic species due to counter-ion condensation effects, and changes in the solvent structure and solvation dynamics. These results provide insight into MTs’ ability to modulate the conductance of aqueous electrolytes, which in turn, has significant implications for biological information processing, especially in neurons, and for intracellular electrical communication in general.

## Introduction

Microtubules (MTs) are hollow cylindrical protein polymers composed of α- and β- tubulin hetero-dimers^1^. These dimers spontaneously self-assemble longitudinally to form protofilaments, and 13 protofilaments constitute a microtubule, resulting in a helical arrangement of tubulin heterodimers. With a diameter of 25 nm, MTs form a rigid structure integral to the cytoskeleton of each eukaryotic cell^2^. Their primary roles include providing a rigid structure to the cell and acting as the wires to pull chromatids apart in mitosis. Also, motor proteins use MTs as a track, with kinesins moving anterograde, towards the positively charged end, and dyneins moving retrograde, towards the negatively charged end of MTs^3, 4^. Kinesin and dyneins play a crucial role in endocytosis and exocytosis, moving organelles, along with chromosome segregation during mitosis and meiosis.

MTs have also been implicated in intra-cellular signalling and information processing, as tubulin has a large dipole moment, and consequently MTs have a large cumulative dipole moment, which provides electrostatic polarity and hence functional directionality^5^. Tubulin dimers which form the MT structure have highly electronegative C-termini, which attract positive counterions, providing a potential mechanism for the observed amplification of ionic signalling^6–8^. It has been hypothesized that MTs are involved in information processing via conductivity effects in neurons as well as in an organism-wide matrix of connected biological wires^9, 10^. As well, exposure to alternating electric fields between 100–300 kHz of strength ~1–2.5 V/cm have been shown to arrest cell mitosis^11^ and have led to an FDA approved treatment of glioblastoma multiforme^12^, with these field effects on MTs being hypothesized as a primary mechanism of action^11, 13, 14^. This latter development provides strong motivation to elucidate the response of solutions with MTs to externally applied AC electric fields.

Potential biological effects of alternating electric fields in the range of hundreds of kHz have recently been reviewed^14^. In particular, dielectrophoretic forces could develop in the presence of an applied field on the order of hundreds of kHz as a result of inhomogeneity in the intracellular electric field and interfere with the proper alignment of the mitotic spindle. Moreover, dipole moment changes in proteins can be induced in the directions of oscillating electromagnetic fields using driving frequencies relevant to protein dipolar relaxation times^15^. One study probed the effect of 10 Hz to 3 kHz AC stimuli on cells transfected with K + channels and on control cells, and found that both kinds of cells oscillated in phase with the driving frequency in the direction normal to the membrane up to frequencies of 1.5 kHz. Because of the sensitivity to the membrane potential and larger amplitude of movement of the transfected cells compared to the wild-type cells, it was hypothesized that the movements of transfected cells were caused by oscillations in the positions of voltage sensor regions of the channels^16, 17^.

Furthermore, there exist controversial claims such as the suggestion that MTs are 1000x more conductive than a single tubulin building block due to the interstitial water channel within the MT^18^. Thus, additional studies of the electrical properties of MTs are warranted, which is one of the reasons for undertaking the research presented in this paper.

**Figure 1:**
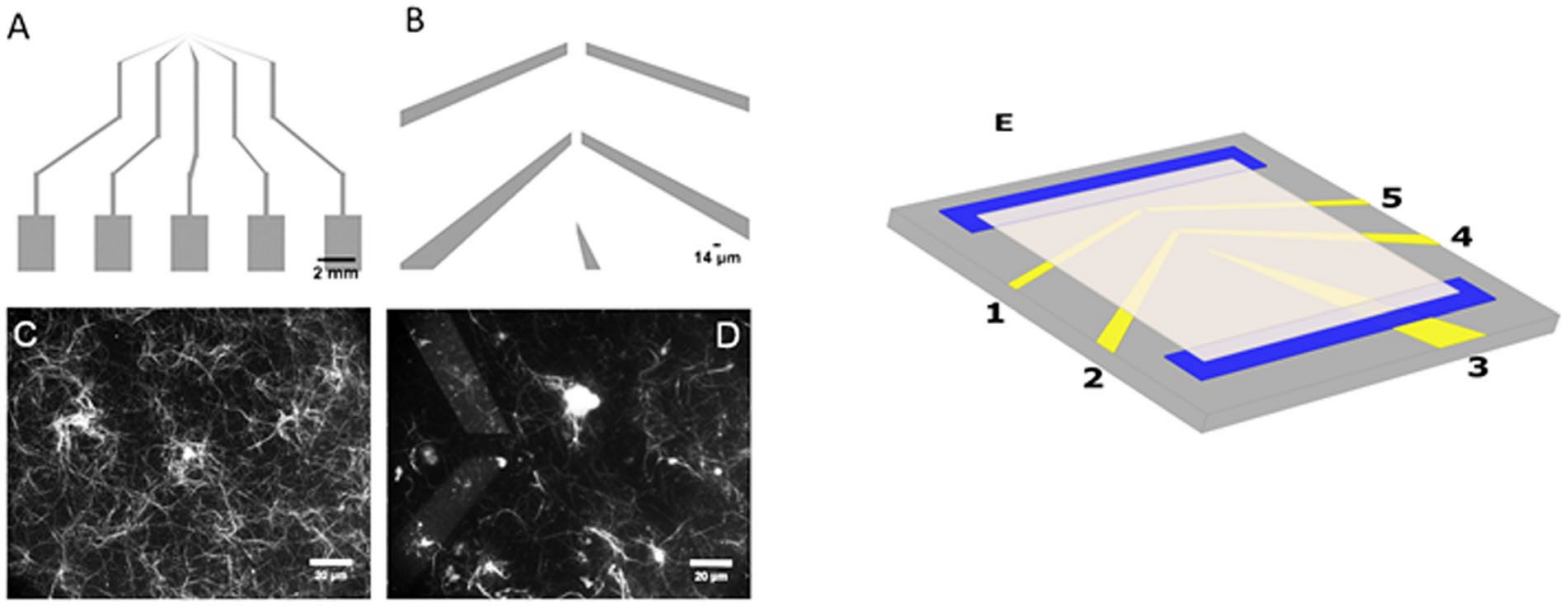
(**A**) Electrode flow cell design used in this study (**B**) Zoomed-in view of flow cell where 5 electrodes converge (**C**) MTs imaged using Zeiss LSM 710 confocal microscope equipped with a 40x objective; excitation wavelength was 592 nm after MT-containing electrolyte was introduced into flow cell; image focused at the top of the flow cell above the electrodes, which are not visible (**D**) Same as (**C**) but image focused on electrode plane (**E**) 3D model of the electrode flow cell in (**A**) and (**B**). 45/10 nm Pt/Ti electrodes (yellow) are printed on the surface of a 1.1 mm thick borosilicate wafer (gray). A 0.15 mm coverslip (white) is placed on top of two pieces of double-sided tape (blue) to create a flow cell with a channel gap of 50 µm width. There is a 14 μm gap between electrodes 2 and 4 which have a length of 30 μm.

**Figure 2:**
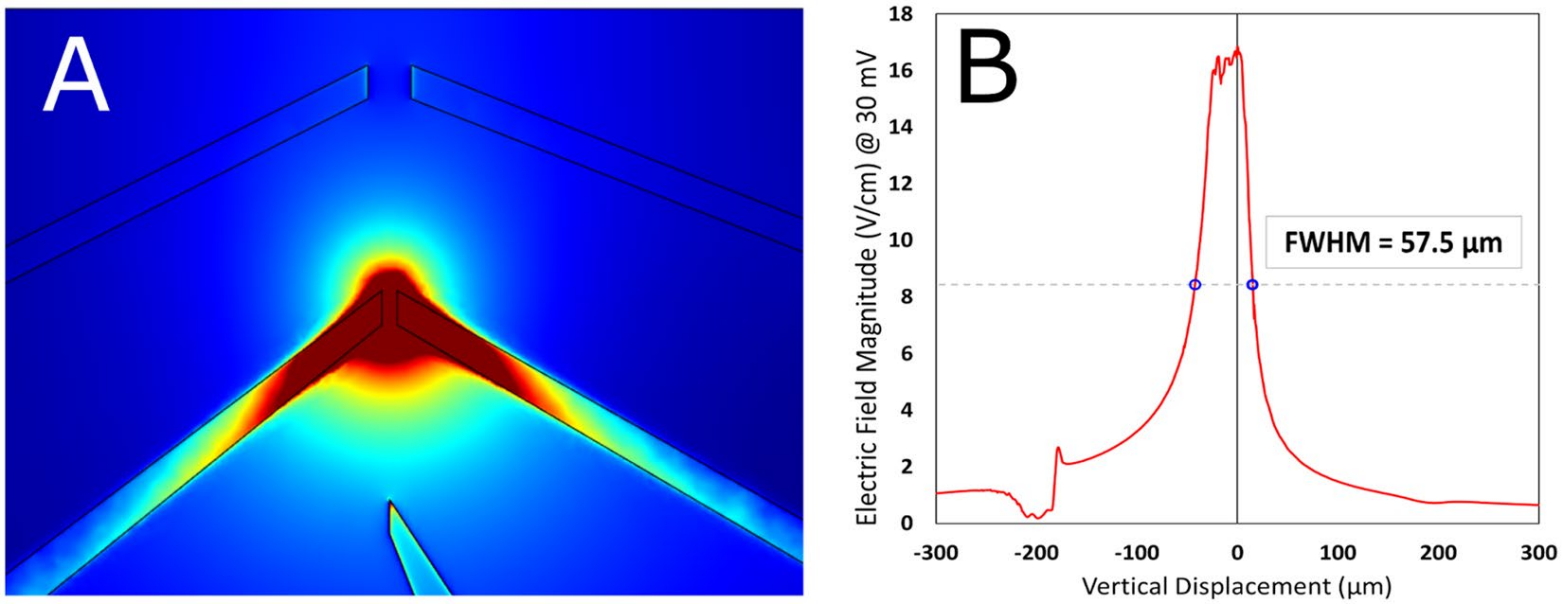
(**A**) COMSOL simulations of the electric field magnitude for a voltage applied between electrodes 2 and 4 (see Fig. 1E) (**B**) Plot of field magnitude along vertical central line between electrodes; FWHM = 57.5 µm.

In this study, we designed two platinum microelectrodes, 14 μm apart (see Fig. 1), and performed electrical characterization of solutions with various concentrations of MTs made of 42.4 nM, 84.8 nM and 212 nM of tubulin, and tubulin dimers, at a range of AC frequencies between 1 kHz and 10 MHz, in a low ionic strength solution. The electric field distribution and relative field strength for the microelectrode geometry employed, are shown in Fig. 2. Microelectrodes offer outstanding performance in eliminating parasitic voltage drops and diffusion effects, and achieving fast equilibration times due to which we utilized them to obtain the conductivity spectra of electrolytic solutions containing tubulin dimers and MT ensembles^19, 20^. The maximum electric field applied was 25 V cm^−1^, for which Faradaic charge injection effects can be ignored in the frequency range studied^21^. Microfluidic impedance spectroscopy, such as used in this work, offers a much higher sensitivity than AC electrokinetic techniques previously used to study the conductivity of MT solutions^8, 22^. A schematic of the cross-section of the flow-cell is provided in Fig. S1. The goal of this study was to quantify the effects of MTs on conductance of solutions, and uncover an optimal protocol to observe MT electrical effects in buffer solutions approaching physiological-like conditions at relevant values of temperature, pH, and ionic concentrations. These results can then be extended to directly study the electrical amplification effects of MTs, which has hitherto been extremely challenging^10^.

## Results

**Figure 3:**
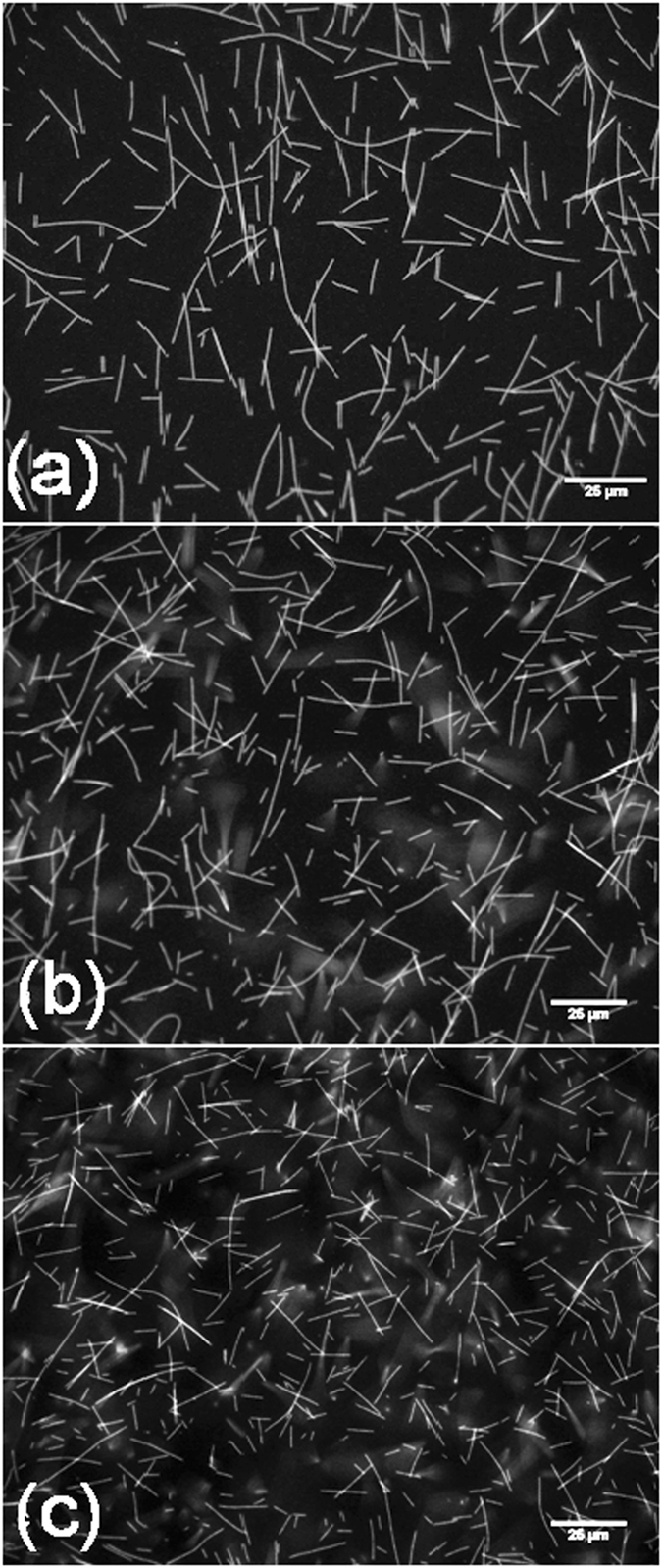
Taxol stabilized MTs remain stable for up to 60 minutes when transferred to a BRB4 buffer. MTs imaged (**a**) 10 minutes after transfer, (**b**) 30 minutes after transfer and (**c**) 60 minutes post-transfer.

MTs are only stable in aqueous electrolytes with a high concentration of solvated ions. MTs spontaneously disassemble in timescales of the order of minutes, in non-aqueous electrolytes and in low ionic strength aqueous electrolytes^23^. The contribution of MTs to the conductivity of high ionic strength buffers is difficult to extract due to the high overall ionic conductance of such electrolytes and the existence of numerous parasitic effects. At the same time, when the ionic strength is decreased below a threshold value in order to uncover the intrinsic conductivity of MTs, the MTs disassemble^23^. These limitations have stymied direct measurements of MT conductance. We determined an optimized protocol that allowed us to overcome the aforementioned competing effects and directly measure the MT contribution to the overall conductance. Figure 3 shows that Taxol-stabilized MTs are stable for at least an hour in the 20-times diluted stock ‘buffer’ electrolyte (BRB4) used by us. Exposure to alternating fields allowed us to understand the frequency dependence of the conductivity and the use of small-signal impedance measurements eliminated the effect of local heating and electrode polarization effects observed at high bias. Previous reports have used poly-L-lysine (PLL) to anchor MTs to a substrate (typically silicon dioxide), in which case the electrostatic interactions of the anchored MTs with the PLL substrate might change the solvation dynamics of the electrolyte ions and MTs^23, 24^. For this reason, MTs were not anchored to the substrate in our studies.

**Figure 4:**
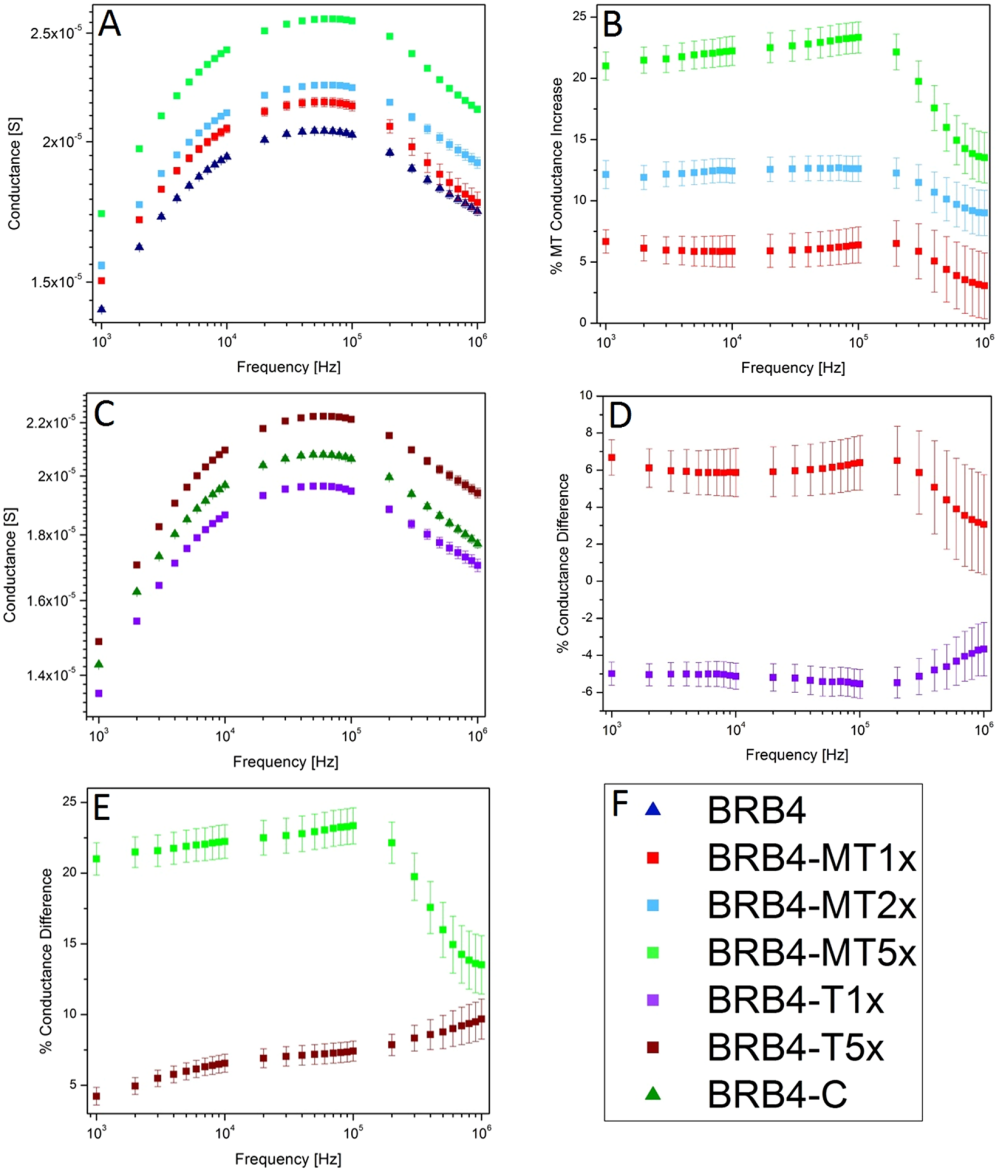
(**A**) Conductance of the BRB4 Buffer (n = 69), BRB4-MT1x (n = 21), BRB4-MT2x (n = 15), and BRB4-MT5x (n = 18) are plotted against frequency (mean, n is the number of trials). Conductance increases with increasing concentration of MTs. (**B**) Mean %-conductance increase for BRB4-MT-(1x,2x,5x) solutions (n = 12, n = 15, n = 18, respectively). (**C**) Conductance of the BRB4 (buffer, n = 24) and BRB4-T1x (un-polymerized tubulin, n = 54) is plotted against frequency. (**D**) BRB4-MT1x (MT solution, n = 12) and BRB4-T1x (unpolymerized tubulin solution, n = 24) %-conductance change vs. BRB4 against frequency. (**E**) %-conductance increase for BRB4-MT5x (n = 18) and BRB4-T5x (n = 24) solutions. (**F**) Legend for Fig. 4A–E.

Our ‘stock’ buffer solution (BRB80) consisted of 80 mM PIPES (pH 6.9), 2 mM MgCl_2_ and 0.5 mM EGTA. BRB80 is a very typical high ionic strength electrolyte in which the MTs are stable for weeks due to which it has been used to conduct investigations of microtubule behavior^25, 26^. In order to distinguish the conductivity contribution of MTs while still maintaining sufficient structural stability of the MTs to enable imaging and electrical measurements, we used an electrolyte we call BRB4 consisting of BRB80 diluted 20 times with milli-Q water. Figure 4 presents the small signal conductance data for electrolytes containing varying concentrations of MTs. Figure 4A shows that MT solutions have a higher conductance than BRB4 buffer alone. As the concentration of MTs increases, so does the conductance of the electrolytes containing them. The conductance increases in the 1 kHz −110 kHz frequency range for BRB4 and BRB4-MT solutions. For frequencies higher than 100 kHz, the conductance begins to decrease for BRB4 and BRB4-MT solutions. In Fig. 4B, the percent increase in conductance for the BRB4-MT solutions compared to BRB4 solution is plotted. As the concentration of MTs increases, the percent increase in conductance follows suit linearly (Fig. S2). The % MT conductance increases slightly from 1 kHz to 100 kHz, and this increase is most pronounced for MTs made using 212 nM of tubulin. Beyond 100 kHz, the % MT conductance begins to decrease slightly for 42.4 nM, 84.8 nM and 212 nM, and this decline is seen most obviously for MTs made from 212 nM of tubulin. In Fig. 4C, it is seen that BRB4 solution has a higher conductance than when free tubulin is added to the solution. The conductance increases from 1 kHz to around 60 kHz for both the BRB4 solution containing colchicine and unpolymerized tubulin, following which the conductance decreases for frequencies higher than 60 kHz. In Fig. 4D, the percent increase in conductance for BRB4-MT1x (MTs made from 42.4 nM of tubulin) and BRB4-T1x (un-polymerized 42.4 nM tubulin) are each compared to BRB4 (buffer) solution. The % conductance increase from MTs made from 42.4 nM of tubulin is similar to the % decrease that 42.4 nM of un-polymerized tubulin results in. From 100 kHz – 1 MHz, both the BRB4-MT1x and BRB4-T1x solutions have their % conductance differences reduced in absolute value.

**Figure 5:**
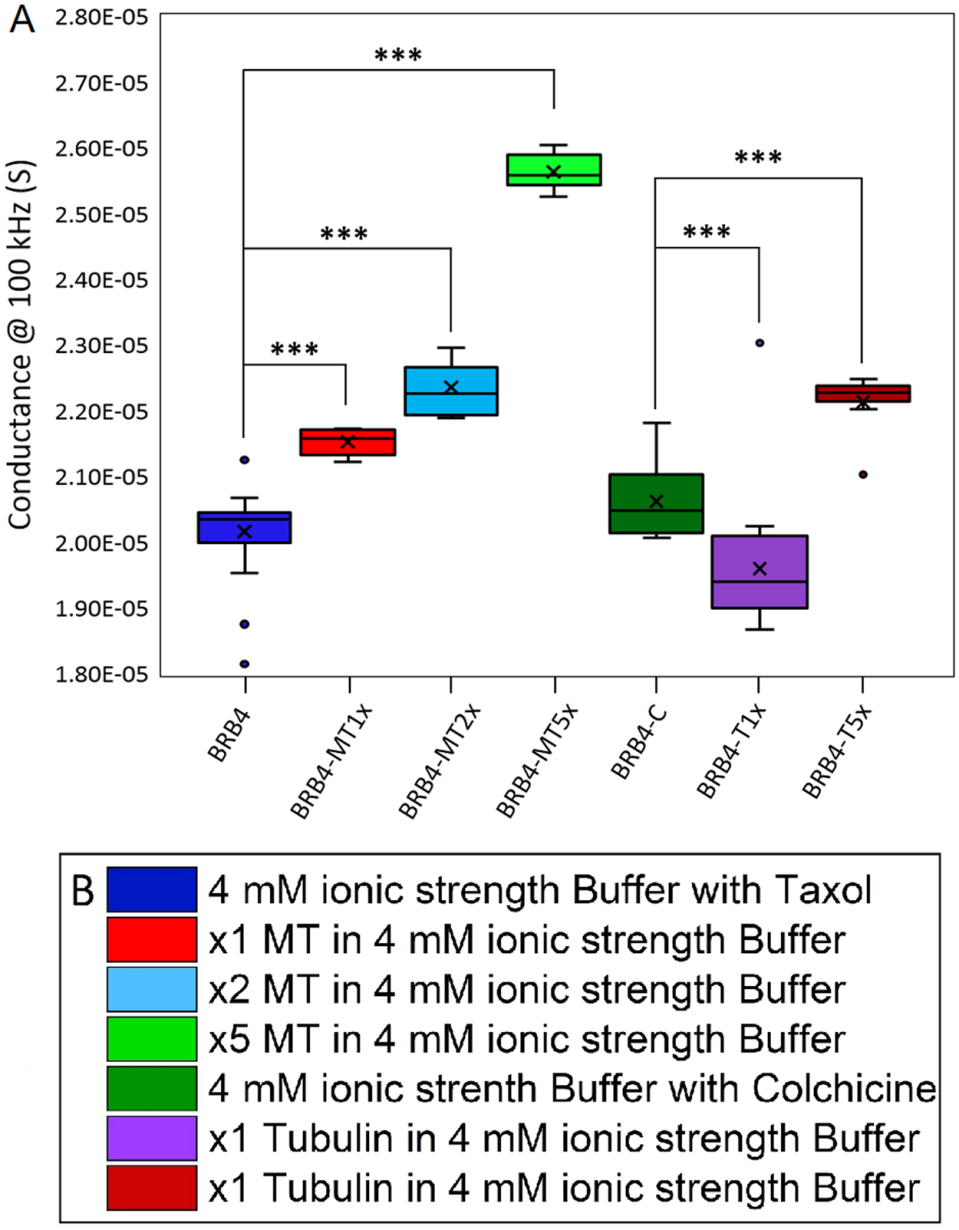
(**A**) Conductance of BRB4 (n = 69), BRB4-MT1x (n = 21), BRB4-MT2x (n = 15), BRB4-MT5x (n = 18), BRB4-T1x (n = 54), BRB-T5x (n = 54), and BRB4-Colchicine (n = 24) at 100 kHz. ***Denotes p < 0.001. (**B**) Legend for Fig. 5A.

Upon adding higher amounts of unpolymerized tubulin to BRB4, the electrolyte conductance experiences a net increase as shown in Fig. 4E. Figure 4E compares the percent increase in conductance for BRB4 solutions with MTs made from 212 nM of tubulin to those containing 212 nM of un-polymerized tubulin each compared to BRB4 solution. The % conductance increase is greater for BRB4-MT5x than BRB4-T5x. From 100 kHz to 1 MHz, the % conductance increase declines for BRB4-MT5x, but still continues to increase from BRB4-T5. The difference in conductance at 100 kHz between BRB4-T1x and BRB4-C is statistically significant (p = 7E-4). The difference in conductance between BRB4 and MT solutions is also statistically significant (BRB4-MT1x, p = 4E-4, BRB4-MT2x, p = 3E-4, BRB4-MT5x, p = 2E-7). As well, the difference in conductance between BRB4-MT1x and BRB4-MT5x is significant (p = 2E-4). Furthermore, the difference between BRB4-MT1x and BRB4-T1x is significant (p = 4E-4). Figure 5 is a histogram plot of the absolute conductance values of the different electrolytes studied, summarizing the concentration-dependent electrical behavior of the tubulin dimer-containing and MT-containing electrolytes in relation to each other and to the low ionic strength buffer (BRB4) electrolyte.

Electric current in a solution of MTs and buffer is modeled as a circuit with distinct parallel resistive elements through which charges can flow. In this case, the total conductance *S*_*tot*_ is:

1 $${S}_{tot}={S}_{B}+{S}_{MT}\Rightarrow \frac{{S}_{tot}}{{S}_{B}}=1+\frac{{S}_{MT}}{{S}_{B}}$$

where *S*_*B*_ is the conductance due to the buffer and *S*_*MT*_ is the conductance due to the MTs. To determine the frequency-dependent characteristics of MT conductance, we assume that the ratio *S*_*MT*_ */S*_*B*_ can be expressed as a function that depends only on frequency *f*:

2 $${S}_{tot}={S}_{B}+{S}_{MT}\Rightarrow \frac{{S}_{tot}}{{S}_{B}}=1+F(f)$$

**Figure 6:**
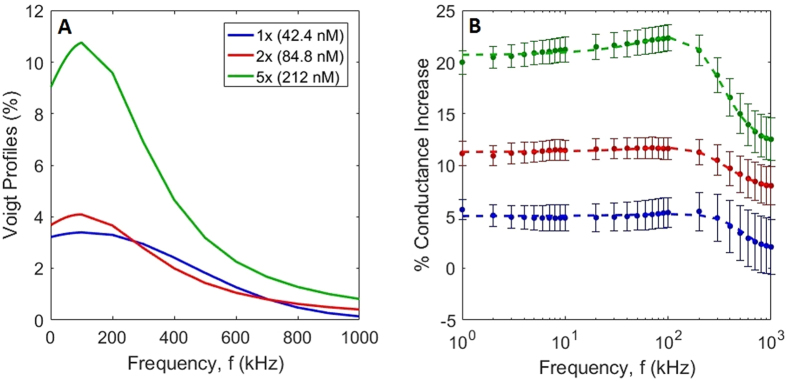
Results of Voigt fitting to measured conductance data using parameters reported in Table 1. (**A**) Background-subtracted Voigt profiles for each MT concentrations using parameters determined in Table 1. (**B**) Measurements (points) and fitting results (dotted lines) of the %-increase of conductance due to the addition of MTs in electrolyte solution.

To characterize the frequency dependence of the observed conductance shown in Fig. 4, we fit the data with a Voigt lineshape. This function is a convolution of a Gaussian and Lorentzian profile:

3 $$F(f)=G(f)\otimes L(f)$$

where *G(f)* is of the form ${A}_{G}\exp (\frac{-{(f-{C}_{G})}^{2}}{2{B}_{G}^{2}})$, and *L(f)* is of the form ${A}_{L}[\frac{{({B}_{L}/2)}^{2}}{{(f-{C}_{L})}^{2}+{({B}_{L}/2)}^{2}}]$, where *A*, *B*, and *C* are the amplitudes, widths, and central frequencies (respectively), and subscripts indicate Gaussian or Lorentzian function parameters. The convolution of these lineshapes incorporates separate broadening mechanisms that are potentially significant in MT-induced conductance variation: The Gaussian profile describes heterogeneous effects such as (1) Doppler broadening that could arise due to the distribution of velocities of the charged species contributing to the measured currents, and (2) the distribution of MT lengths along which charged species travel. Conversely, the Lorentzian describes homogeneous broadening mechanisms such as collisional losses due to interacting Coulomb fields. The choice of the Voigt lineshape allows for natural interpretations of amplitude *A*, width *B* (FWHM), and central frequency *C* that can be related to the observed conductance behaviours of MTs. There is an additional background offset parameter that represents a frequency-independent increase of conductance due to the addition of microtubules. Optimal parameters were determined by weighted least-squares analysis using the measured data and values predicted by Eqs 2 and 3. The results of these optimizations are given in Table 1. Plots of the profiles corresponding to these parameters are shown in Fig. 6A, and the comparison to the measured data is shown in Fig. 6B.

**Table 1:** Results of fitting measured conductance data to Voigt profiles.

*Relative MT Concentration*	*Amplitude A (%)*	*Width (FWHM) B (kHz)*	*Central Frequency C (kHz)*	*Conductance Offset (%)*	*R* ^*2*^
1x	3 ± 7	809 ± 1448	116 ± 98	2 ± 2	0.9794
2x	4 ± 2	586 ± 288	100 ± 17	8 ± 2	0.9928
5x	11 ± 2	503 ± 118	111 ± 11	12 ± 2	0.9991

**Figure 7:**
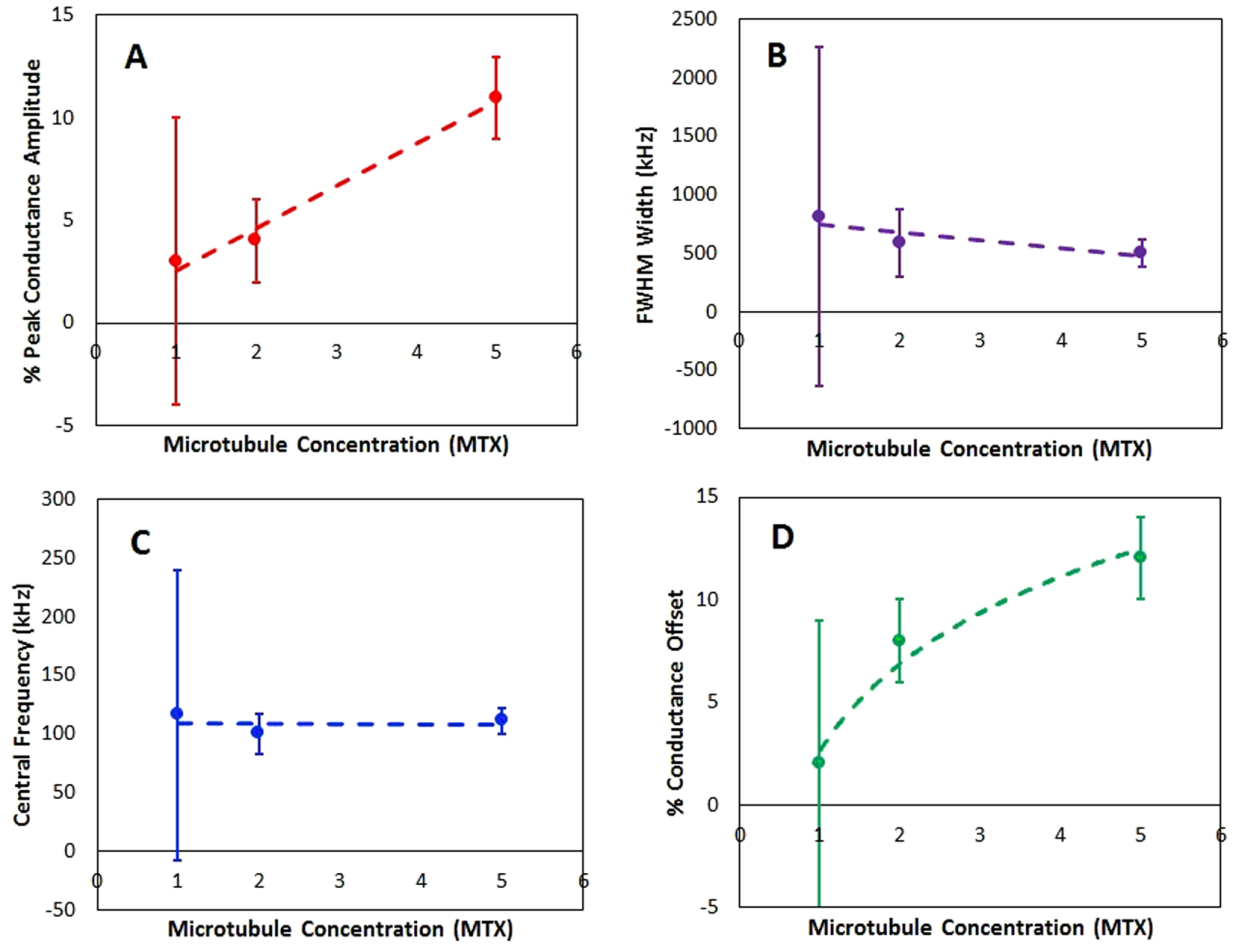
Variation of extracted parameters with relative concentration of MTs (**A**) Amplitude of peak conductance (**B**) Width of the conductance peak (**C**) Central frequency corresponding to maximum conductance and (**D**) % Conductance offset.

These fit parameters provide several insights to the observed characteristics in the measured conductance ratio spectra. The background conductance offset represents a frequency-independent increase due to the addition of MTs to the buffer solution. The amplitudes *A* in Table 1 indicate an additional {3, 4, 11} % conductance increase for {42.4, 84.8, 212} nM MT solutions, representing a linear increase with concentration that can be attributed to applying a voltage at an optimal frequency for charge transport (Fig. 7A). The frequency that this peak conductance increase occurs at is independent of MT concentration (Fig. 7C), and the linearly decreasing profile width (Fig. 7B) therefore indicates increasing “quality” of the conductance spectrum as MT concentrations increase. This peak behavior was not observed in the buffer solution alone (fits not shown), and so this peaked conductance response at a preferred frequency is interpreted as an intrinsic property of the MTs in aqueous electrolytes. Further, due to the close correspondence between the Voigt and Lorentzian lineshapes (not shown), we believe the dominant broadening mechanism to be homogenous (i.e. collisional). The discussed observations are consistent with the hypothesis of electrical conductivity oscillations, and we therefore do not reject the possibility that the observed effects could be explicitly described as resonant-like behaviour of a damped oscillator. However, further studies are required to precisely determine the physical mechanism behind the characteristic features of these lineshapes.

## Discussion

The effects of MTs and unpolymerized tubulin on the small-signal AC conductance and capacitance of a low ionic strength buffer solution (BRB4) were measured (see Section S1 and Fig. S3 for a discussion of the electrolyte strength and the effect of dilution on the buffer conductance). In the 1 kHz to 1 MHz range, MTs increased the measured conductance, and this effect was linearly dependent on the concentration of MTs added to the solution. MTs at concentrations 42 nM, 85 nM, and 212 nM tubulin increased solution conductance by *ca*. 6, 11, and 23%, respectively at 100 kHz. Conversely, unpolymerized tubulin at 42 nM concentration lowered the measured conductance relative to BRB4 by *ca*. 5% at 100 kHz. The methodology reported in this paper provides a robust way of measuring the conductance effects of MTs in solution. We found that the molar conductance contribution of MTs peaked in the range of 80–300 kHz and was quantified to be ~10^5^ Sm^2^ mol^−1^.

The AC conductivity behaviors exhibited by the electrolytes investigated in this study are typically explained by the MT ionic transmission wire hypothesis wherein counter-ions attracted by MT C-termini and ions in the lumen of MTs have a higher ionic mobility, as modeled previously^6, 27^. The ability of weakly-bound counter-ions to diffuse along the long axis of the MTs without tight constraints is critical to the increase in ionic mobility^8^. However, for the values of applied field used in this study, simultaneous imaging of MTs while bias was applied (not shown), indicated no alignment or orientation of the MTs across the electrodes in the direction of the applied field i.e. there were no continuous paths through the MTs between the electrodes. Furthermore, the highest concentration of MTs used was ~6 × 10^8^ MTs μL^−1^, at least six orders of magnitude lower than the concentration of ions. Consequently, the ionic transmission wire hypothesis is not relevant to our study. We attribute the conductivity increase of MT-containing electrolytic solutions to the effect of MTs on solvent structure and solvation dynamics. Both tubulin-dimers and microtubules contribute to the asymmetric solvation of ions since each tubulin monomer has a charge of −47e and is surrounded by an atmosphere of neutralizing cations^28^. It is also well known that the structure of bound water around macromolecules is different from that in the bulk, with disordered regions between the bound and bulk water^29^. As well, it has been found that the structure of water changes at the border between solutions and bulk materials^30^. A secondary reason for the observed electrical behavior is provided by the polarization dynamics of solvated charged species. According to the classical solvent-berg model, the polarization cloud of solvent dipoles around each ion is assumed to be rigidly bound to the respective ions, resulting in a decrease in the ionic mobility (*μ*_*i*_) due to the increased hydrodynamic radius (*r*_*i*_) of the new unit comprising the ion and the bound dipoles per the expression^31^:

4 $${\mu }_{i}^{\infty }=\frac{{z}_{i}{F}_{i}}{6\pi {N}_{A}\eta {r}_{i}}$$

where *N*_*A*_ is the Avogrado number, *F* is the Faraday constant, *η* is the solution viscosity and *z* is the charge on the ion.

The high cumulative dipole moment of tubulin dimers and MTs^32, 33^ can modify the polarization behavior of the electrolyte containing them, and weaken the polarization of solvent dipoles around mobile ions, thus reducing the effective hydrodynamic radius in Eq. (4) and resulting in a higher ionic mobility. An interesting finding in our work is that, although BRB4-T1x decreased solution conductance relative to BRB4, we found that BRB4-T5x increased solution conductance, but at a lower amount compared to BRB-MT5x. That is, when tubulin is added to the buffer solution at sufficient concentration, the conductance increases. The opposing electrical behaviors of low concentration tubulin dimer-containing BRB4-T1x (decrease in ionic conductance vs. BRB4-C) and high concentration tubulin dimer-containing BRB4-MT5x (increase in ionic conductance vs. BRB4-C) can be reconciled as follows: unpolymerized tubulin may decrease ionic mobility, due to its larger molecular size and due to the condensation of counter-ions around the surface of tubulin. For higher tubulin concentrations, the decrease in the hydrodynamic radius of ions and concomitant increase in ionic mobility due to screening of Coulombic interactions becomes more significant.

We presented a conductance model of damped oscillators to account for the increase of conductance found in solutions containing MTs, with the goal of better understanding the frequency dependence of the relative conductivity of the BRB4 solution and that of MTs, i.e. *σ*_MT/_ *σ*_B_. *σ*_BRB4_ was measured to be 0.05 S m^−1^. Several caveats must be mentioned with our results. The measurements of MTs increasing the conductance of the solution shows a bulk MT effect. In the biological cellular environment, MTs would exist in an ordered cytoskeletal network. Our reported effects are in a low ionic environment, which was done to more clearly discern the MTs’ effects on conductance. However, our results point to MTs amplifying the ionic mobility, and this may be the case in a wide range of ionic concentrations.

Interestingly, the conductance of MT-containing electrolytes peaked at 80–300 kHz, which overlaps with the range of “Tumor Treating Fields” (TTFs) used to treat glioblastoma-multiforme^12^. Our modelling predicts that this could be due to frequency-dependent electrical oscillations, as the conductance increase was found to be broadly peaked at ~ 100 kHz. In the presence of an alternating electric field at a sufficiently high frequency, the field oscillations may occur on a shorter time scale than the time required to build the asymmetric ionic polarization cloud. By eliminating the relaxation effect, this may lead to a higher conductance of the buffer solution, and explain the sharp decrease in conductance difference for high frequencies, seen in Fig. 6B. In other words, the observed conductance peak may be related to a plasma frequency of the counter-ion polarization cloud loosely bound to the MTs. This may, at least partially, account for the observed effects in the cellular environment where the TTField assays were conducted.

## Conclusion

MTs were found to lead up to a 23% higher conductance when added to BRB4 solution, while unpolymerized tubulin led to 5% lower conductance at lower concentrations (1X) and a 5% higher conductance at higher concentrations (5X). Hitherto, the electrical properties of MTs have been primarily viewed in the context of their behavior as ionic transmission nanowires. By examining the behavior of MTs in lower ionic strength electrolytes using a microelectrode flow cell, we show that MTs increase the conductivity of electrolytic solutions that contain them independent of, and in addition to, the ionic transmission wire hypothesis. We attribute the higher conductivity of MT-containing electrolytes to counter-ion condensation and the effects of MTs on solvent structure and solvation dynamics. Further experiments to test the role of MT and actin in electrical communication of neurons and in an intracellular matrix are warranted. The conductance contribution of MTs peaked at 95–110 kHz, in a similar range as that of TTFields, which may help explain the mechanism of action of TTFields.

## Methods

### Solution preparation

Rhodamine-labeled fluorescent MTs were polymerized from 5 µL of porcine brain tubulin (Cytoskeleton kit #BK004R). Tubulin (Cytoskeleton, Kit #BK007R) was prepared following the protocol for the Fluorescent MTs Biochem Kit, with parameters used to obtain long MTs. Briefly, we followed these steps:

One aliquot of rhodamine tubulin was resuspended in 4.25 µl BRB80 (microtubule buffer solution aka PEM, 80 mM PIPES, pH 6.9, 2 mM MgCl_2_ and 0.5 mM EGTA (Cytoskeleton, BST01)) and 0.75 µl MT Cushion Buffer (5% [v/v] glycerol) to yield 4.0 mg/ml fluorescent tubulin. 16 µl of unlabeled tubulin (5.0 mg/ml) was added to the 5 µl of fluorescent tubulin (4.0 mg/ml), leading to 21 µl of tubulin (1 rhodamine: 3 unlabelled tubulin; 4.8 mg/ml). Four 5-µl aliquots were made per rhodamine tubulin aliquot. An MT solution was prepared by polymerizing 5 µl tubulin aliquot prepared as stated above supplemented with 0.2 µL GTP (100 mM) at 37 °C for 30 min, followed by adding 100 µl PEM solution containing Taxol (10 µM), yielding an 850 nM tubulin solution of fluorescent MTs in BRB80. 1x ProLong Gold anti-fade mountant (ThermoFisher scientific, P36930), was added to MT solutions to be imaged. MTs using Nikon A1r confocal microscope equipped with motorized Prior XY stage and Z drive. 25x water immersion objective (DIC N2) was used for microtubule visualization; excitation wavelength was 592 nm. Microtubule solutions were imaged prior to experimentation to confirm their presence before all experiments.

### BRB4 solution (buffer)

To study the conductance of MTs, we used a low ionic solution to minimize ionic contribution to conductivity. Low ionic states have previously shown MTs’ increased conductivity^8, 22^. Thus, we used BRB4 ionic strength solutions. BRB4 ionic strength buffer was prepared by adding 5 μL of BRB80 buffer (80 mM PIPES pH 6.9, 2 mM MgCl_2_ and 0.5 mM EGTA (Cytoskeleton, Inc. BST01) to 95 μL Milli-Q water.

### BRB4-MT1x solution

BRB4-MT1x solutions (42.4 nM tubulin) for testing were prepared by adding 5 μL of MT solution (850 nM tubulin in BRB80) to 95 μL Milli-Q water.

### BRB4-MT2x solution

BRB4-MT2x solutions (84.8 nM tubulin) were prepared by adding 5 μL of MT2x solution (1.7 μM tubulin in BRB80) to 95 μL Milli-Q water.

### BRB4-MT5x solution

BRB4-MT5x (212.5 nM tubulin) were prepared by adding 5 μL of MT5x solution (4.25 μM tubulin in BRB80) to 95 μL Milli-Q water.

### BRB4-T1x solution

Unpolymerized tubulin was prepared by incubating a 5 μL aliquot of tubulin supplemented with 20 μM colchicine at room temperature for 30 minutes, at which point 100 μL of PEM was added to the protein solution to yield the BRB4-T1x solution. The BRB4-T1x solution was then prepared by diluting 5 μL of the stock tubulin solution (850 nM tubulin in BRB80) in 95 μL of Milli-Q water.

### BRB4-T5x solution

The BRB4-T5x stock solution was prepared in the same fashion as BRB4-T1x however the starting volume of protein solution was 25 μL (with 20 μM colchicine) and only 75 μL PEM was added to the solution following incubation at room temperature for 30 minutes to yield a final solution volume of 100 μL. Prior to testing BRB4-T5x solution (212 nM tubulin in BRB80) was prepared by adding 5 μL of BRB4-T5x stock solution (4.25 μM tubulin in BRB80) to 95 μL of Mili-Q water.

### BRB4-C solution

PEM buffer supplemented with 20 μM colchicine to make the BRB4-C stock solution. Prior to testing 5 μL of BRB4-C was diluted in 95 μL of Milli-Q water to yield the working buffer solution that was used for testing.

### Device Fabrication

For electrical characterization, platinum (Pt) electrodes were fabricated on a square 10 cm borofloat wafer (Swiftglass) using standard photolithography processes. Photoresist (HP504) was spincoated (500 rpm – 10 s, 4000 rpm – 40 s) and patterned as a mask for subsequent metal evaporation and liftoff. Microwire electrodes of thickness 55 nm (10 nm Ti/45 nm Pt) were evaporated, followed by acetone liftoff. Fisher coverslips (#1 18 × 18 mm^2^; Fisher Scientific 12–542 A) were mounted on the wafer using Scotch^®^ double-sided sticky tape (3 M) to create a flow cell. The device used consisted of two electrodes 30 µm wide, separated by 14 µm (see Fig. 1).

### Electrical Characterization

Conductance and capacitance frequency sweeps were performed using a semiconductor characterization system (CVU-integrated Keithley 4200-Semiconductor Characterization System) with a probe station. The range of applied frequencies was from 1 kHz to 10 MHz, which is the largest range of frequencies that the semiconductor characterization system can apply, with logarithmic step sizes. Each sweep took 8 seconds. Probes were placed on wires 2 and 4. Multiple voltage sweeps were performed in each experimental situation (the number of sweeps done is reported in the figures).

The flow cell was flushed with BRB4. Subsequently, three conductance frequency sweeps were performed. The flow cell was then flushed with BRB4-MT1x solution, and three conductance frequency sweeps were performed. The same protocol was used for BRB4-MT2x, BRB4-MT5x, BRB4-T1x, and BRB4-T5x solutions.

Measurements were performed at room temperature, and the maximum Joule heating of the samples due to AC stimulation was estimated to be negligible (on the order of ~1 µK).

### Conductivity Meter Testing

BRB4 was made by adding 0.5 mL of BRB80 to 9.5 mL of mili-Q water. An HM Digital COM-100 EC/TDS/Temperature Meter was submerged into the BRB4 solution and swirled around. After waiting for one minute for the reading to stabilize, the conductivity of the BRB4 solution was recorded.

### Calculations

Conductance (S) reported is the mean of all frequency sweeps. The number of sweeps for each figure is reported in the figure caption. % MT Conductance Increase is calculated using the following formula:

5 $$\delta =\frac{{S}_{MT}-{S}_{Buffer}}{{S}_{Buffer}}$$

Error is sample standard deviation.

## Electronic supplementary material

Supporting Information
