# Supplementary material for: Response to Alternating Electric Fields of Tubulin Dimers and Microtubule Ensembles in Electrolytic Solutions
Source: Sci Rep. 2017 Aug 29;7:9594. doi: 10.1038/s41598-017-09323-w (PMC5574899; doi:10.1038/s41598-017-09323-w)
Supplement: Supplementary file 1 — Supporting Information [file 41598_2017_9323_MOESM1_ESM.pdf]

Supplementary Information

Response to Alternating Electric Fields of Tubulin Dimers and Microtubule Ensembles in Electrolytic Solutions

Iara B. Santelices[1,2], Douglas E. Friesen[2], Clayton Bell[2], Cameron M. Hough[2,5], Jack Xiao[1,2], Aarat Kalra,[1,2,4] Piyush Kar[1], Holly Freedman[6], Vahid Rezania[7], John D. Lewis[2], Karthik Shankar[1,3] and Jack A. Tuszynski[2,4]

1. Department of Electrical & Computer Engineering, University of Alberta, Edmonton, Alberta T6G 1H9, Canada
2. Department of Oncology, University of Alberta, Edmonton, Alberta T6G 1Z2, Canada
3. NRC National Institute for Nanotechnology, Edmonton, Alberta T6G 2M9, Canada
4. Department of Physics, University of Alberta, Edmonton, Alberta T6G 2E1, Canada
5. Department of Medical Physics, Cross Cancer Institute, Edmonton, Alberta T6G 1Z2, Canada
6. Department of Medical Microbiology and Immunology, University of Alberta, Edmonton, Alberta T6G 2E1, Canada
7. Department of Physical Sciences, Grant MacEwan University, Edmonton, Alberta T5J 4S2, Canada

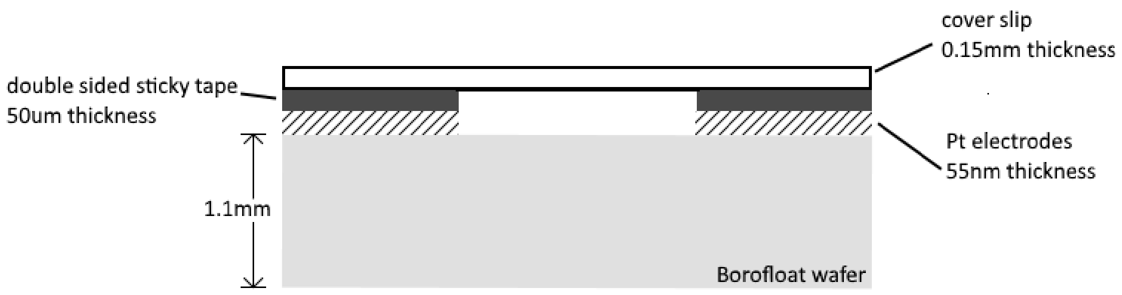

Figure S1. Side-view (right) of the flow cell used for electrical characterization.

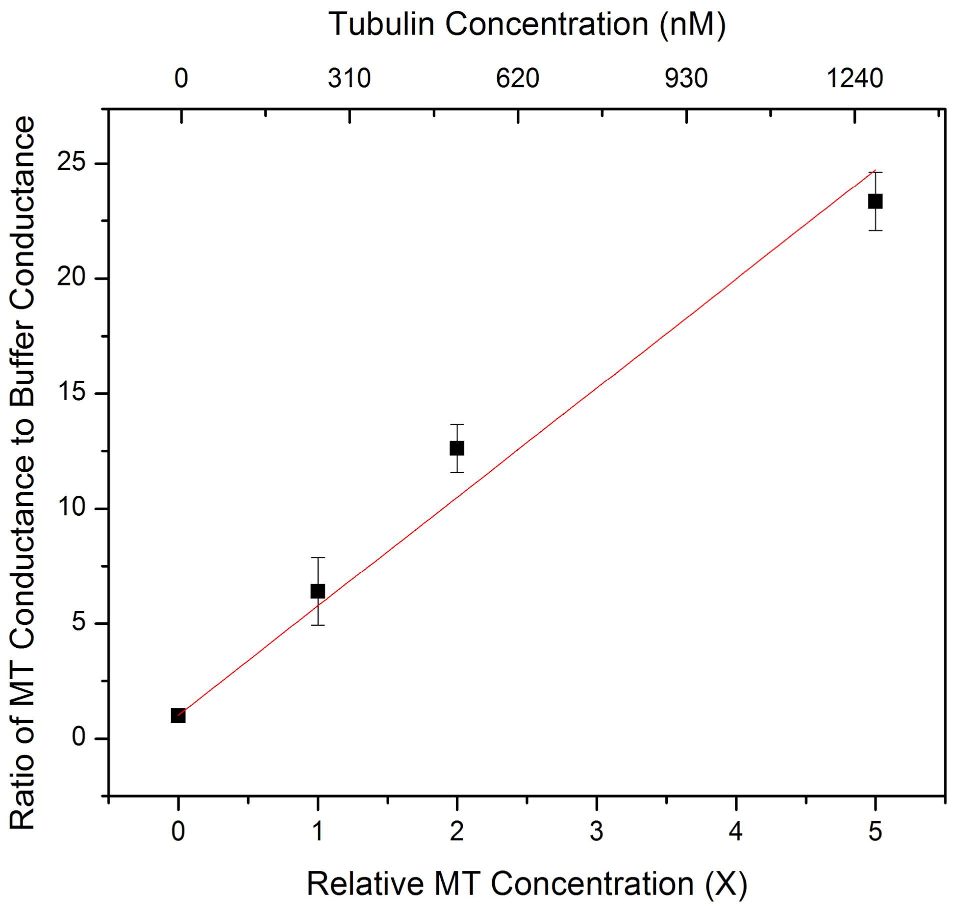

Figure S2. Plot of the ratio of the conductance of MT-containing BRB4 electrolytes vs. MT concentration, and linear fit to the same.

Section S1. Molar conductance of buffer

In Fig. S3A we plot the dependence of the buffer conductance S_{buffer} on the concentration of PIPES (which is approximately half the concentration of sodium ions in the buffer). The molar conductance S_{buffer}/c_{stoich} is plotted against ionic concentration in Fig. S3B. Non-ideality of the electrolyte i.e. decrease in molar conductance with increased ionic concentration may be observed. Two well-known factors generally contribute to such non-ideality, known as the relaxation effect and the electrophoretic effect. Typically a solvated ion attracts oppositely charged ions into its surrounding ionic atmosphere. However when an ion moves in the direction of an applied electric field, there are more like-charged ions than oppositely-charged ions ahead of it, and more oppositely-charged ions than like-charged ions behind it, and this asymmetric ionic atmosphere slows the central ion down. The time required for a new ionic atmosphere to be formed around a central ion is known as the relaxation time, and this retardation effect is known as the relaxation effect. Moreover, in the presence of an external electric field, since negatively charged and positively charged ions are moving in opposite directions and each pulls along with it a polarized solvent shell (solvent-berg model), when these ions cross paths there is a viscous drag effect on both ions as a result of the interaction between the solvent shells of the oppositely charged ions, known as the electrophoretic effect. In addition, for a weak electrolyte that is not fully dissociated, the fraction ionized c_{actual}/c_{stoich} may increase dramatically as the stoichiometric concentration c_{stoich} approaches 0, leading to a sharp increase in molar conductance, and this may also contribute to the non-ideality seen in Fig. S3B.

From Debye-Huckel theory [1], the molar conductivity **Λ** of a symmetrical strong electrolyte solution and thus molar conductance as well, varies approximately linearly with the square root of c_{stoich}:

$$\Lambda = \Lambda^0 - \text{constant}\sqrt{c_{stoich}} \qquad (0)$$

Here $\mathbf{\Lambda} = \kappa/c_{stoich}$, where **κ** is the conductivity and $\mathbf{\Lambda^0}$ is the limiting molar conductivity. We examined the fit to this expression of our measured values of buffer conductance. As shown in Fig. S3C, we determined an R^2 value of 0.90 for the linear correlation between $\sqrt{c_{stoich}}$ and **Λ**. The buffer solution used by us satisfied Kohlrausch's law (Eqn 0) for strong electrolytes (see Fig. S3C) but the steep slope of the fit indicated behavior in-between strong and weak electrolytes. Therefore, it is expected that at lower PIPES buffer concentrations, the correlation may no longer hold and the electrolyte most likely dissociates much better at lower concentrations. Thus the limiting molar conductivity of the buffer $\mathbf{\Lambda^0}$ is likely underestimated by the y-intercept of the fit line in Fig. S3C.

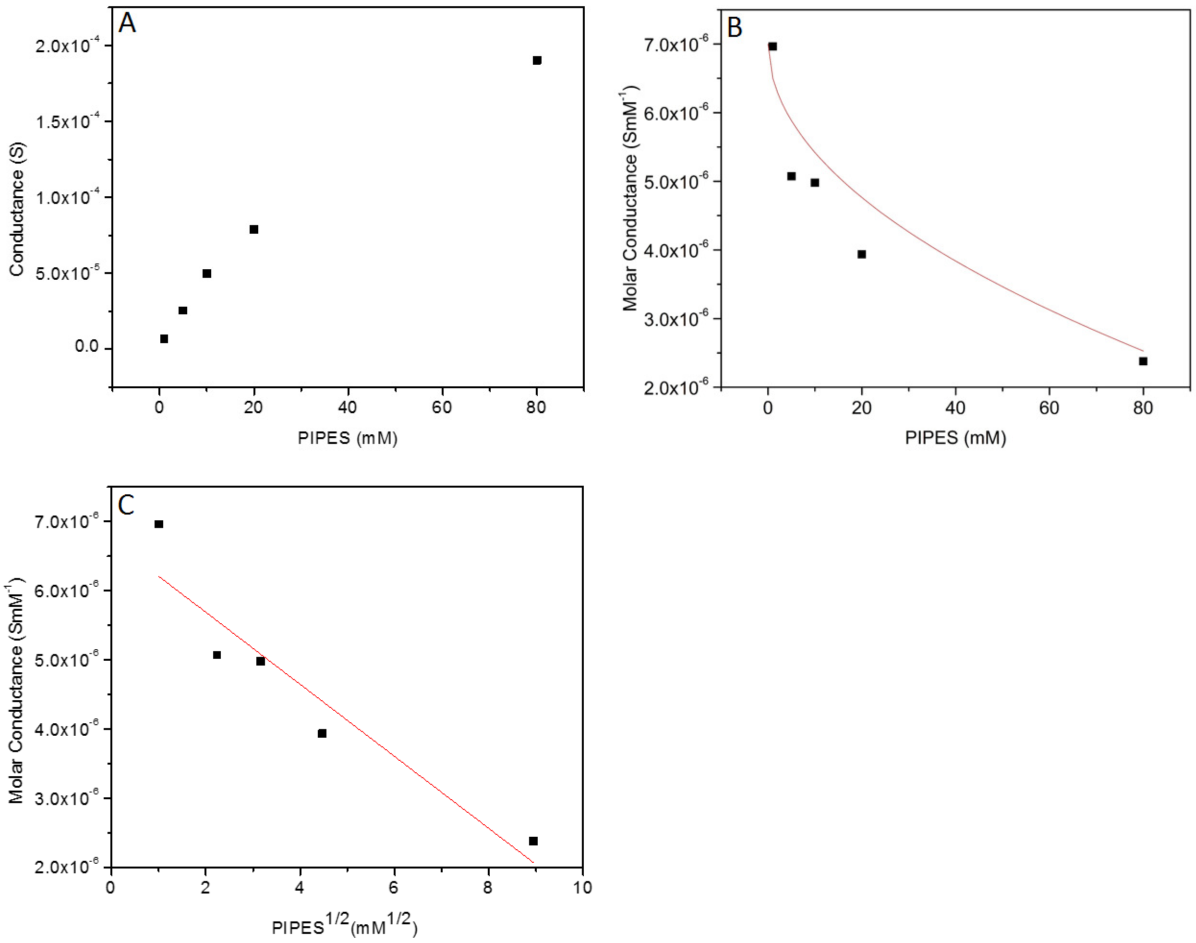

Figure S3. **(A)** Conductance and **(B)** molar conductance of buffer solution plotted against the concentration of PIPES in mM, averaged over a sample size of n=3 **(C)** Average molar conductance of buffer solution plotted against the square root of the concentration of PIPES in $mM^{1/2}$ (b), at a frequency of 100 kHz. Discrete points represent experimental data points and lines represent best fits to equation 0.

Section S2. Capacitance variation in microtubule ensembles

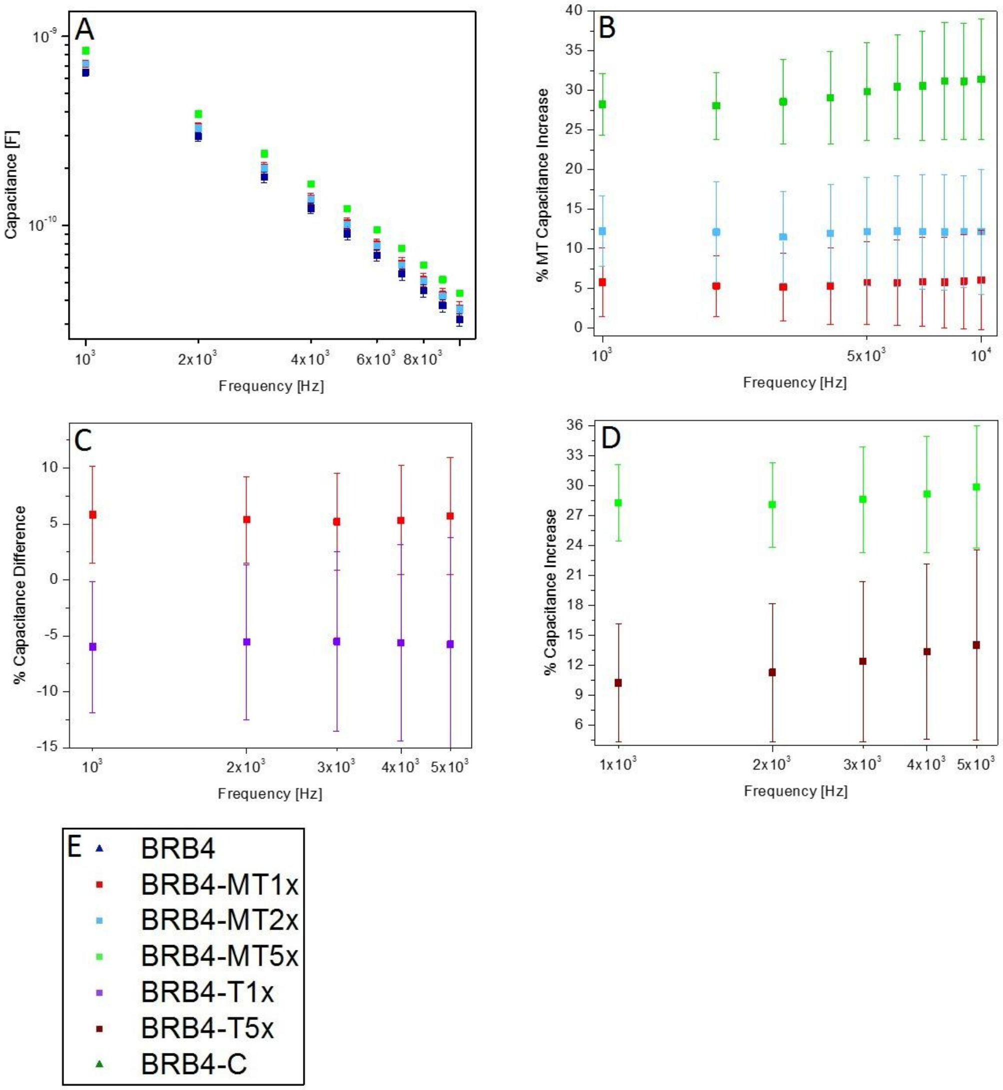

Figure S4. (A) Capacitance values of the BRB4 Buffer (n=69), BRB4-MT1x (n =21), BRB4-MT2x (n=15), and BRB4-MT5x (n=18) are plotted against frequency (mean, n is the number of trials). **(B)** Mean %-capacitance increase for BRB4-MT-(1x,2x,5x) solutions (n=12, n=15, n=18, respectively). **(C)** BRB4-MT1x (MT solution, n=12) and BRB4-T1x (unpolymerized tubulin solution, n=24) %-conductance change vs. BRB4 against frequency. **(D)** %-capacitance increase for BRB4-MT5x (n=18) and BRB4-T5x (n=24) solutions. **(E)** Legend for figures 4 A-E.

Section S3. System transfer function

We performed further analysis of our experimental data by determining the transfer function of the system. The transfer function for the system was determining by first finding the appropriate equivalent circuit for the system, which was derived from frequency-dependent resistance versus reactance plots (i.e. Nyquist plots). For microtubules (and tubulin) suspensions in buffer, as well as bare buffer solutions,

the applicable equivalent circuit is shown in Fig. S5.

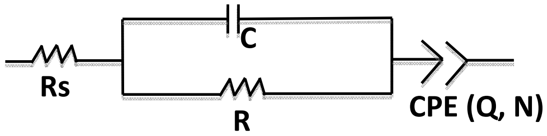

Figure S5. Illustration of the equivalent circuit for the system, which is similar to that reported in literature for a similar system [2].

In Fig. S5, resistive circuit element, **Rs,** is be attributed to a parasitic resistance effect due to the platinum electrodes and cables. The other elements are a time-constant given by a capacitance (**C**) parallel to a resistance (**R**), and a constant phase element (**CPE**). In case of microtubule solutions, the capacitance **C** is the capacitance of microtubules in parallel to the capacitance of solvated ions in the solution. In case of buffer, the **C** is the net capacitance of ions in the buffer. Similarly, in case of microtubule solutions, **R** is resistance of parallel combination of resistances of microtubules and that of the buffer. In case of buffer, the **R** is the resistance of the buffer. The constant phase element **CPE** has a magnitude **Q** and a coefficient **N**. **Q** is a measure of the concentration of ions that are close to the electrode-electrolyte interface and increases with the strength of the buffer solution[1], and **N** is the fractal dimension related to electrode surface roughness[1].
Defining:

$$\mathbf{W_i = 2.\pi.F_i} \tag{1}$$

In (1)**,** **i** is the step count of frequency, **i** was considered between 1000 to 50,000 Hz.

By definition:

$$\mathbf{Z_C = \frac{1}{jW_i.C}} \tag{2}$$

$$\mathbf{Z_{CPE(Q,N)} = \frac{1}{(jW_i)^N.Q}} \tag{3}$$

In (2) and (3) **j** equals to $\mathbf{(-1)^{0.5}}$. The transfer function of the equivalent circuit, mentioned in Fig. 1, is given by (4). **Z** is the total impedance of the system. The real part of the transfer function is denoted by **Z'** and the imaginary part is denoted as **Z"**. A plot of **Z'** versus **- Z"** gives the Nyquist plot. The Nyquist plot fit of experimental data (as shown in Fig. S6) yielded the values of **Rs**, **C**, **R**, **Q** and **N**, which are given in Table S1.

Table S1. Values of fitting parameters of the above-mentioned equivalent circuit

	**Rs (Ohms)**	**C (F)**	**R (Ohms)**	**Q ($\mathbf{Fs^a}$)**	**N**
**MT1x**	4.50e4	1.20e-8	6.20e3	2.40e-7	0.620
**MT2x**	4.38e4	1.20e-8	5.20e3	2.30e-7	0.620
**MT5x**	3.82e4	1.20e-8	4.70e3	2.30e-7	0.620

**Buffer**	4.85e4	9.50e-9	8.50e3	2.40e-7	0.665
**Colicane Buffer**	4.75e4	9.20e-9	8.50e3	2.30e-7	0.620
**Tx1**	5.03e4	9.60e-9	6.60e3	2.00e-7	0.620
**Tx5**	4.45e4	9.60e-9	6.60e3	2.20e-7	0.620

$$Z = Rs + \frac{R}{1 + W_i^2 R^2 C^2} + \frac{W_i^N}{Q}.\cos(N.\pi/2) - j\frac{C}{1 + W_i^2 R^2 C^2} - j\frac{W_i^N}{N}.\sin(N.\pi/2) \quad (4)$$

a = -1+N;

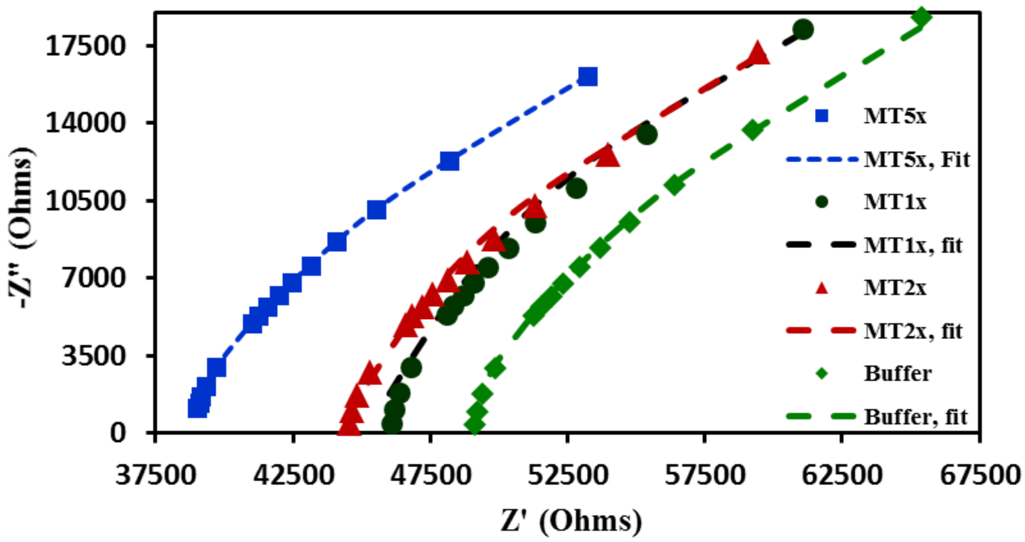

Figure S6. Nyquist plot showing experimental and fit curves for MT1x, MT2x, MT5x and buffer.

Considering the parallel combination for resistances of microtubules and buffer in buffer containing microtubule suspensions, resistance of microtubules can be calculated by using (5).

$$R_{MT} = \frac{R_{MT,buffer} , R_{buffer}}{(R_{buffer} - R_{MT,buffer})} \quad (5)$$

Table S2. Resistance and capacitance values evaluated from the impedance spectroscopy data by assuming resistance and capacitance of microtubules and tubulin to be parallel to those of buffer

	Resistance (Ohms)	Capacitance (F)
**MT1x**	2.29e4	2.50e-9
**MT2x**	1.58e4	2.50e-9
**MT5x**	1.05e4	2.50e-9
**Tx1**	2.95e4	1e-10
**Tx5**	2.95e4	1e-10

In (5), $\mathbf{R}_{\mathbf{MT}}$ is the resistance of microtubules (or tubulin), $\mathbf{R}_{\mathbf{MT,buffer}}$ is the total resistance of microtubules (or tubulin) containing buffer, and $\mathbf{R}_{\mathbf{buffer}}$ is the resistance of buffer without microtubules (or tubulin). Table S2 lists the resistance values of MT5x, MT2x, MT1x, T1x and T5x, obtained using (5).

For capacitance, we may consider the capacitance of microtubules (or tubulin) to be in parallel with that of solvated ions in buffer. Then capacitance of the microtubules (or tubulin) may be calculated by using (6).

$$\mathbf{C}_{\mathbf{MT}} = \mathbf{C}_{\mathbf{MT,buffer}} - \mathbf{C}_{\mathbf{buffer}} \tag{6}$$

In (6), $\mathbf{C}_{\mathbf{MT}}$ is the capacitance of microtubules (or tubulin), $\mathbf{C}_{\mathbf{MT,buffer}}$ is the total capacitance of microtubules (or tubulin) containing buffer, and $\mathbf{C}_{\mathbf{buffer}}$ is the capacitance of the buffer without microtubules (or tubulin). Table S2 lists the capacitance values of MT5x, MT2x, MT1x, T1x and T5x, obtained using (6).

Section S4. Frequency response of common electrolytes in the microelectrode geometry used

In high ionic strength electrolytes, the admittance spectra exhibit the expected behavior of microelectrode arrays in the form of the conductance increasing linearly with frequency in the studied range (1 kHz - 10 MHz); this behavior is manifested for all the electrolytes studied, namely BRB80, Phosphate Buffered Saline (PBS) solution, 10 mM KCl, 10 mM NaCl, 100 mM KCl and 100 mM NaCl (Figure S7). The lower-than-bulk conductance of the microelectrodes at lower frequencies derives from the polarization of the electrodes, which is specific to a particular microelectrode geometry as shown in Green *et al*, 2000 [3].

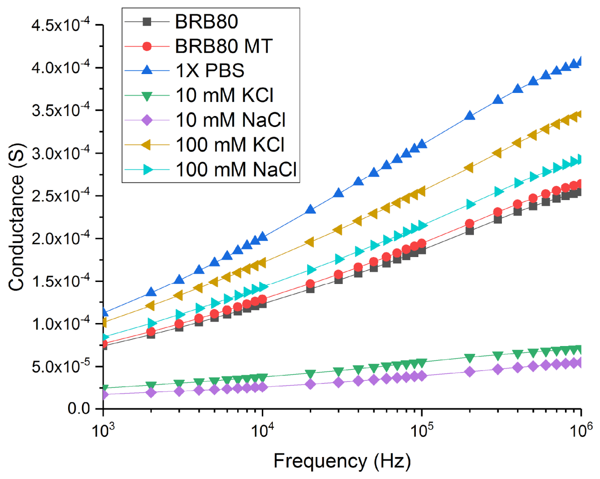

Figure S7. Variation of conductance as a function of frequency for various electrolytes in the microelectrode geometry used

Analysis of our data at 4 mM electrolyte concentrations reveal that conductance, which is low at frequencies in the kilohertz range, increases with frequency. Once frequencies in the megahertz region are explored, however, conductance reduces. This behavior, discussed in Jadżyn *et al*, 2015 [4], can be explained due to two effects. At low frequencies, conductance is small due to the presence of the double layer capacitor at the electrode surface, which leads to damping and reduces the effective potential gradient in the cell. As the frequency is increased, the double layer capacitor becomes more conducting and the electrolyte conductance correspondingly increases. At frequencies higher than the plasma frequency ω_p [5-7], ions in the double layer are unable to keep pace with the rapidly switching electric field, and thus reduce the net conductivity of the solution.

In summary, the two effects that cause non-ideal behavior of the frequency-dependent electrolyte conductance are the microelectrode geometry (which increases the relative importance of the double layer capacitance on the surfaces of the electrodes) and the low ionic concentration of the electrolytes used in this study. The effect of higher ionic concentrations is to shift the conductance maximum to higher frequencies, as demonstrated in Figs. S8 (A) and (B) below, due to the increase in the plasma frequency. Because the maximum frequency in our measurements was 1 MHz, this peak in conductance is not present for high ionic strength solutions such as 100 mM NaCl, 100 mM KCl, and BRB80. While the effect of the double layer capacitor still exists in the low frequency range, the ion inertia effect is compensated by the increased ion concentration.

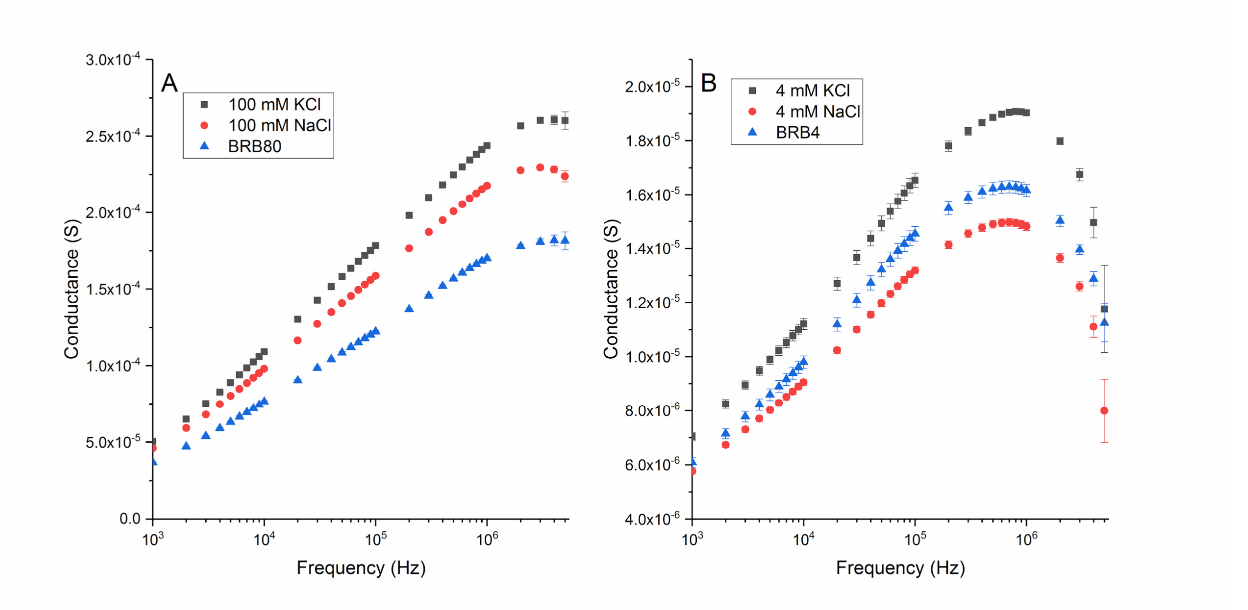

Figure S8 (A) Variation in the conductance as a function of frequency for 100 mM KCl, 100 mM NaCl and BRB80, and (B) Observed peak in conductance as a function of frequency for 4 mM electrolyte concentrations.

References

[1] M. R. Wright, *An Introduction to Aqueous Electrolyte Solutions*: John Wiley & Sons, 2007.

[2] H. Sanabria, J. H. Miller Jr, A. Mershin, R. F. Luduena, A. A. Kolomenski, H. A. Schuessler, and D. V. Nanopoulos, "Impedance Spectroscopy of α-β Tubulin Heterodimer Suspensions," Biophysical Journal, vol. 90, no. 12, pp. 4644-4650, 6/15/, 2006.

[3] N. G. Green, A. Ramos, A. González, H. Morgan, and A. Castellanos, "Fluid flow induced by nonuniform ac electric fields in electrolytes on microelectrodes. I. Experimental measurements," Physical Review E, vol. 61, no. 4, pp. 4011-4018, 2000.
[4] I. Płowaś, J. Świergiel, and J. Jadżyn, "Frequency dependence of the ionic conductivity in water + ammonium nitrate electrolyte solutions," Electrochimica Acta, vol. 178, pp. 511-516, 10/1/, 2015.
[5] V. I. Gaiduk, B. M. Tseitlin, and J. K. Vij, "Orientational/translational relaxation in aqueous electrolyte solutions: a molecular model for microwave/far-infrared ranges," Physical Chemistry Chemical Physics, vol. 3, no. 4, pp. 523-534, 2001.
[6] T. Dodo, M. Sugawa, E. Nonaka, and S.-i. Ikawa, "Submillimeter spectroscopic study of concentrated electrolyte solutions as high density plasma," The Journal of Chemical Physics, vol. 116, no. 13, pp. 5701-5707, 2002.
[7] V. Thakore, and J. J. Hickman, "Charge Relaxation Dynamics of an Electrolytic Nanocapacitor," The Journal of Physical Chemistry C, vol. 119, no. 4, pp. 2121-2132, 2015/01/29, 2015.
